# Predicting Engagement Patterns With Connected Wearable Devices in a Health System: Survival Analysis

**DOI:** 10.2196/78507

**Published:** 2025-09-17

**Authors:** Allistair Clark, Gillian Gresham, Joshua Pevnick, Raymond Duncan, Mitchell Kamrava, Michael Sobolev

**Affiliations:** 1 Department of Medicine Cedars-Sinai Medical Center Los Angeles United States; 2 Behavioral Design Unit Cedars-Sinai Medical Center Los Angeles, CA United States; 3 Enterprise Information Services Leadership Group Cedars-Sinai Medical Center Los Angeles, CA United States; 4 Radiation Oncology Academics Cedars-Sinai Medical Center Los Angeles, CA United States

**Keywords:** wearable electronic devices, remote patient monitoring, electronic health record, patient engagement

## Abstract

**Background:**

The rapid advancement and widespread adoption of wearable devices provide opportunities to collect longitudinal objective activity and health data and integrate the information directly into a patient’s electronic health record (EHR). Patterns of engagement and factors associated with the use and nonuse of wearable devices are currently not well understood.

**Objective:**

This study aimed to quantify the number of patients still engaged and using wearable devices at 1 year since each patient’s first day of use across a cohort collected over 6 years. We then aimed to identify demographic and behavioral factors that statistically significantly predict the likelihood of staying engaged and using wearable devices within the same 1-year time span since first use.

**Methods:**

We analyzed connected device data from a large, nonprofit academic medical center, which began to incorporate wearable device data into the EHR system in April 2015. We conducted a survival analysis to evaluate time to early disengagement among connected device users and identify factors associated with long-term (1 y) engagement in multivariable Cox proportional hazard regression models.

**Results:**

The analysis included 8616 patients (mean age 45, SD 14.36 y; median 21, IQR 34-55 y; men: n=4489, 52.1%; women: n=4126, 47.9%) with available connected device data (eg, step counts) from the EHR between 2015 and 2022. A total of 5870 (68.13%) patients were engaged with active connected devices in the EHR at 1 year. Multivariable Cox regression models indicated no statistically significant differences between gender groups and race categories. Younger age categories (18-34 y) and lower median daily step counts (<5000) were associated with statistically significant increased hazards for early disengagement at 1 year.

**Conclusions:**

The ongoing development of new sensors and algorithms presents opportunities to expand the capabilities of wearable devices, making them even more integral to health care delivery. It is important to quantify and enhance engagement to maximize the benefits of this technology and inform future use of the technology to improve health outcomes.

## Introduction

### Background

Wearable technology has rapidly evolved into a valuable tool in health monitoring and management [1]. Over the last decade, consumer-facing wearable devices have become increasingly popular, sophisticated, and accurate [2,3]. Currently, more than one-third of US adults use smartwatches or fitness bands, with nearly 80% of device owners willing to share their data with medical providers [4]. Contributing to Apple’s popular consumer technology base, recent years have seen a notable increase in wearable adoption with the release of the Apple Watch (Apple, Inc). These devices enable real-time collection of activity and health data, offering transformative opportunities for monitoring, diagnosing, and managing a wide range of health conditions if implemented successfully into standard health care workflows [1].

An extensive body of literature now exists, demonstrating the clinical validity of data measured by modern wearable devices by linking the wearable device data to a range of health outcomes [5-7]. For example, a recent meta-analysis reported that taking more steps per day was related to a reduced risk of all-cause mortality [8]. Other studies have established a negative relationship between the number of steps taken and cardiovascular disease–related mortality [7] as well as the odds of hospitalization and survival of patients with cancer [6,9]. These findings highlight the clinical value and potential applications of wearable devices in enabling continuous monitoring outside clinical settings, informing personalized treatments, and playing a role in early detection [1]. Furthermore, step count can also serve as an indicator of engagement with physical activity and devices that promote it.

Health care systems have started to explore the connection between wearable devices and the integration of wearable device data into the electronic health record (EHR) [10,11]. For example, a review from 2019 identified at least 16 health systems that currently deploy these systems [11], with adoption continuing to increase. Data from wearable devices contribute to the strengthening of the ecosphere of medical devices in health care, often called the Internet of Things, which enhances health care delivery, diagnostics, and patient care through advanced data collection and communication across a wide variety of platforms and systems [1]. However, numerous barriers associated with the implementation of wearable health technology exist and are likely to hinder the clinical and research value of these data [11,12].

One challenge is ensuring sufficient patient engagement with wearable devices so that data can be useful for medical research and clinical practice. In research, differential attrition and engagement in remotely collected health data can lead to nonrandom missing data, affecting generalizability. In clinical practice, missing data may undermine decision-making and support tools dependent on them. Therefore, recent research is increasingly focusing on engagement, as exhibited by use, adherence, and attrition to wearable device adoption. For example, a depression study found that approximately 68% of the patients continued to share passive Fitbit (Google LLC) data after 43 weeks, even after many of them stopped actively engaging with the study app and other study components [13]. However, engagement in the real-world setting often differs significantly from the study setting or self-reported data [14]. Therefore, there is a need to investigate objective and long-term engagement with wearable devices outside of the controlled research setting.

### Objectives

The purpose of this study was to quantify engagement with commercial wearable devices in the health care setting and identify patient-level drivers of long-term engagement and disengagement. In April 2015, our health care system began integrating wearable health data into the EHR and invited patients to synchronize their consumer-directed health wearable devices (eg, Fitbit and Apple Watch) directly through the Epic MyChart patient portal. Building on previous research on initial engagement with digital technology linked to our health system, we operationalized engagement with wearable devices as frequency of use, as indicated by the daily report of step count to the EHR, with disengagement defined as the last recorded daily step count observation per patient [13,15]. To investigate patient engagement with wearable devices, we compiled wearable engagement data from January 2015 to January 2022 that included 8616 patients from the health care system. We examined engagement, defined as sustained device connection over a 1-year period, within this cohort and studied the impact of demographic and objective behavioral factors associated with early disengagement.

## Methods

### Study Design

This retrospective, longitudinal, observational study was conducted at a large academic health system in Los Angeles, California, which integrated device data into the EHR via the Epic MyChart–powered patient-facing portal. Epic is the largest provider of EHR software in the United States. We analyzed device data, defined as any consumer-based device (eg, wearable activity tracker and phone) used to track activity, connected to the EHR between January 1, 2015, and January 1, 2021.

### Participants

This study included the complete sample of patients who were aged 18 years or older during the period in which any of their wearable health devices were connected to EHR. Patients received a 1-time instruction in MyChart on how to connect their wearable devices, with methods varying by device brand. Fitbit and Withings users linked their wearables through the web-based MyChart page, authorizing direct vendor-cloud transfers into Epic. Apple and Google Fit users completed linkage in the mobile MyChart app, which pulled daily feeds from the third-party Apple HealthKit or Google Fit apps, which in turn were linked to the patients’ devices. Apple HealthKit also included data from linked third-party devices, such as Kardia (AliveCor), Omron, and Oura. HealthKit data were collected until January 1, 2022, to allow for sufficient follow-up. Multiple same-day data were aggregated into daily averages per patient.

### Recruitment and Setting

This is a retrospective, longitudinal study using passively collected data from the EHR, where no direct patient contact or active recruitment was required. We used all available cases that met the predefined eligibility criteria with at least 1 wearable device linked to the EHR-based workflow between 2015 and 2021 within the aforementioned large academic health system in Los Angeles, California, United States. All records were deidentified and filtered to retain only patients who were aged 18 years or older at the time of their first device connection. Upon ethics review, participant consent was not required, given the retrospective nature of the study, deidentified data, and alignment with the study’s objectives, as described further in the Ethical Considerations section.

### Ethical Considerations

This study obtained ethics approval from the Cedars-Sinai Institutional Review Board (STUDY00002591). A waiver of informed consent and Health Insurance Portability and Accountability Act authorization was granted based on the study’s classification as minimal risk, the absence of direct interaction with participants, and the likelihood of bias or loss of statistical power due to attrition or death if consent were required. The collection of identifiable contact information for obtaining consent would have exceeded the scope of this retrospective secondary analysis.

All data used in this study were fully deidentified before analysis. No identifiable personal information was retained, and no reidentification was attempted. Consequently, privacy and confidentiality protections were in place, consistent with research involving human participants. Because this study did not involve direct recruitment or interaction with participants, and consent was waived, no compensation was provided. No images or multimedia appendices include identifiable individuals. If future versions of this manuscript include such materials, appropriate consent will be obtained, and documentation will be provided.

### Variables

The available EHR extract contained 61 unique variables, including demographics (ie, age, gender, race, and ethnicity), wearable device metadata (ie, types of devices connected and date that the wearable device was first linked), and wearable device data corresponding to the health metric recorded (type of measurement, measurement value, date the measurement was recorded on device, date the measurement was uploaded to the EHR framework, and the type of device). We identified 7 variables of interest: gender, age, race, type of wearable device metric, measurement value of the health metric, the date the measurement was recorded by the device, and device type (eg, Fitbit or Google Fit, Apple Watch or iPhone, or other device).

### Statistical Analysis

This is an observational study that includes all patients who connected wearable devices to the EHR. Therefore, no formal hypotheses that necessitate power calculations were defined. The study objectives were to measure time to disengagement and identify patient sociodemographic factors associated with early disengagement, defined as a user having their last day of observation in the entire dataset within their respective 365-day follow-up window. This per participant last day of observation within a 1-year follow-up period indicates a device disconnect from the refined EHR database and therefore represents a patient’s disengagement from the system. . Exploratory analyses were conducted to evaluate the distribution of the included data, including patient demographic data (age, gender, and race) and device data (eg, steps/day). Outliers identified as artifacts (eg, duplicate same-day values, infeasibly large health metric outliers, or observations in the first and last percentile) were removed as well as any observations that were manually entered by the patient into the EHR (Figure S1 in Multimedia Appendix 1 provides the full process of data cleaning). In addition, a major update to the EHR device connection workflow rendered observations in 2022 noncomparable to previous observations. Consequently, all data from January 1, 2022, and onward were deleted. Furthermore, patients who had less than 7 days of observations in the entire dataset were excluded from the final analyses. Finally, step count observations at or below the first percentile were also excluded, as such low step counts are more likely due to erroneous measurement caused by not wearing the device or its improper fit. This included all patients and associated data who connected a device after January 1, 2021, as they did not have a sufficient follow-up window and would bias further analyses. Descriptive data, including means (SD) for continuous variables, such as age and step count, and percentages for categorical variables, such as gender and device type, were then generated.

Missing data were minimal after initial exclusion criteria were applied; however, any remaining missing values for key covariates (eg, age, gender, and race) were listwise deleted for multivariable modeling. Sensitivity analyses were conducted to assess the robustness of findings under alternative follow-up definitions, including a 3-month window and follow-up from the first observation to the last valid recorded measurement within the dataset. Results varied minimally from the findings of the 1-year follow-up window. Consequently, a 1-year follow-up period was chosen to ensure stable estimation of attrition while also offering a standardized, interpretable time frame for evaluating engagement—unlike variable-length follow-up based solely on data availability.

To quantify engagement with wearable devices over time, we conducted a time-to-event analysis, with device disengagement being defined as the event. For these analyses, we defined an event as the patient’s last daily step count report, thus indicating device disengagement from the EHR framework. Kaplan-Meier curves were generated to evaluate connected wearable device engagement by demographic variables, including gender, age at first observation, and year of patient sign-up (year of first observation). We also categorized patients’ median first 7-day step counts following the most common method for such analysis [16] and included these in the models. These factors were then included as covariates in a multivariable Cox proportional hazards model to identify factors associated with device disengagement within this observational data set. For all analyses, we defined the follow-up period as 1 year (365 days) from the patients’ first observation. Patients who had no observations on or after day 365 (eg, day 366 and onward) were considered to have had their devices disengaged from the system and thus marked as experiencing the event. All analyses were carried out in RStudio (version 4.2.3; Posit PBC), using the *survival* and *survminer* packages. The proportional hazards assumption was assessed for this model using the cox.zph function from the *survival* R package and found to be nonsignificant.

## Results

### Overview

The final dataset consisted of 8616 patients with a mean age of 45.09 (SD 14.36) years. In total, 4489 (52.1%) identified as men, and 5368 (62.3%) patients were White (Table 1). The observations in the refined dataset used for this investigation consisted of daily step count metrics. This metric was chosen due to its ubiquitous presence across the dataset, consisting of half of all observations in the original dataset.

**Table 1:** Demographic characteristics of patients included in the final analysis dataset (N=8616).

Characteristic		Values, n (%)
**Gender**		
	Man	4489 (52.1)
	Woman	4126 (47.89)
	Nonbinary	1 (0.01)
**Race**		
	Asian	886 (10.28)
	Black or African American	908 (10.54)
	White	5368 (62.3)
	Other	715 (8.3)
	Unknown	739 (8.58)
**Ethnicity**		
	Hispanic	1228 (14.25)
	Non-Hispanic	6699 (77.75)
	Unknown	689 (8)
**Age (y)**		
	Value, mean (SD)	45.09 (14.36)
	Value, median (IQR)	21 (34-55)
**Steps**		
	Value, mean (SD)	5427.87 (4360.01)
	Value, median (IQR)	5642 (2095-7737)

Table 1 displays the key demographic characteristics of the finalized sample of patients who connected a wearable device between 2015 and 2021 that was used in this study’s analyses. This table includes age at first device connection, gender, race, ethnicity, and average number of steps taken within the first 7 days of device connection.

### Device Type

Initial exploration of the dataset indicated that patients historically connected 4 distinct device brands: Apple HealthKit, Fitbit, Google, and Withings (with 9020 unique devices). Across the refined dataset, the 2 most prevalent wearable devices connected by patients were Apple and Fitbit (Table 2), followed by Withings and then Google Fit.

**Table 2:** Count of unique device types connected to the electronic health record from 2015 to 2022 (n=9020)^a^.

Devices	Count, n (%)
Apple HealthKit	7496 (83.1)
Fitbit	1279 (14.18)
Withings	191 (2.12)
Google Fit	54 (0.6)

^a^Some IDs may be connected to multiple devices. Minimal same-day multiple device measurements occurred in 89 of the 2,436,946 observations.

**Figure 1:**
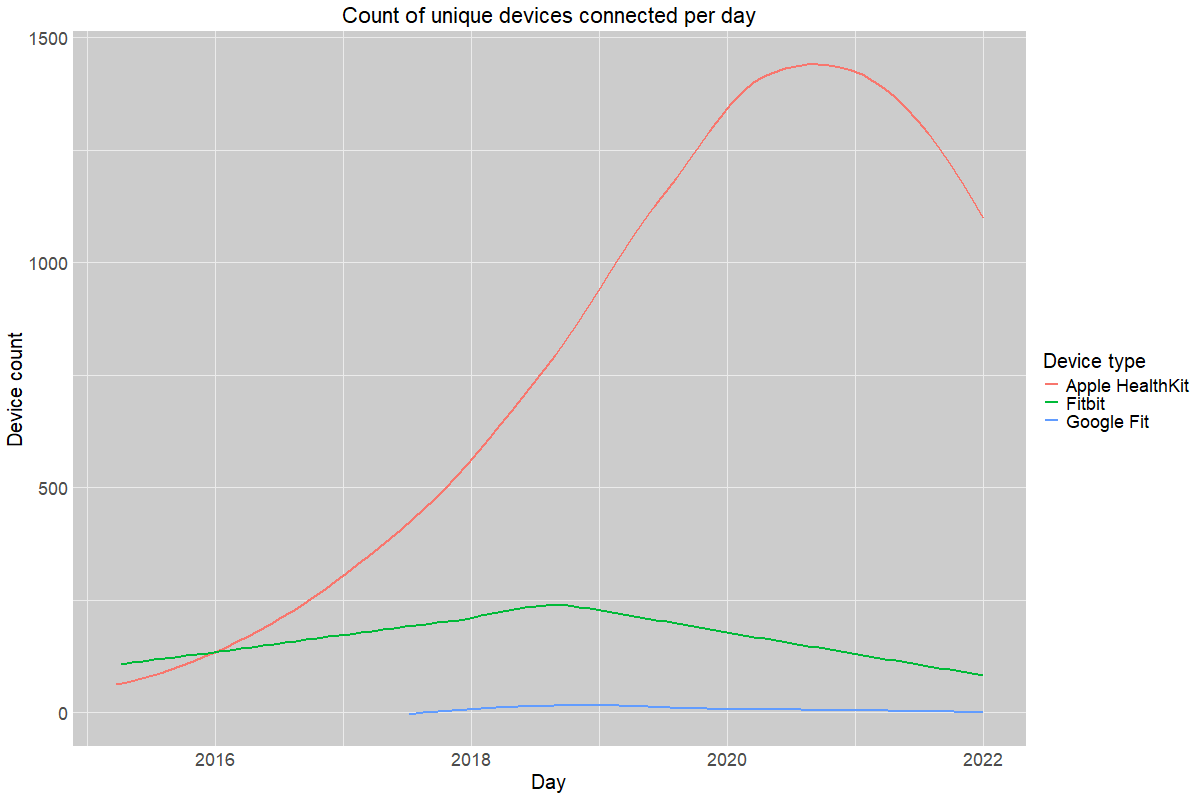
Count of unique step count–recording devices connected to the electronic health record framework between 2015 and 2022.

Figure 1 represents the daily count of unique devices uploading step counts to the EHR framework by overall device type. The red curve depicts Apple HealthKit uploads, the green curve depicts Fitbit uploads, and the blue curve depicts Google Fit uploads.

Device type prevalence is unequally distributed across the data. Figure 1 depicts the aggregate count of devices signed up per month across different device types (Apple HealthKit, Fitbit, and Google Fit) over the entire observation window. From 2016 until the end of observations, Apple HealthKit notably outpaced all other device sign-ups. Of note, while records indicate that patients had Withing devices linked at any point during their interaction with the workflow, no valid step observations originated from this brand of device.

### Time to Device Disengagement

**Figure 2:**
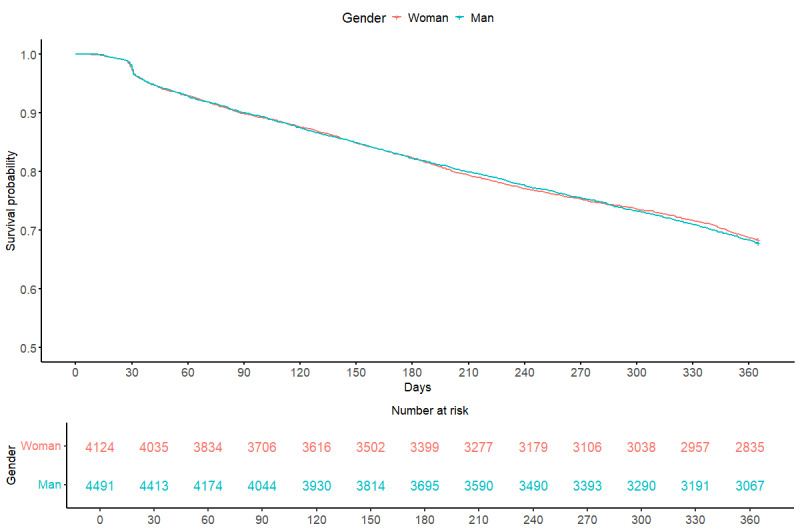
Time to device disengagement among 8616 connected patients over a 1-year period by gender.

**Figure 3:**
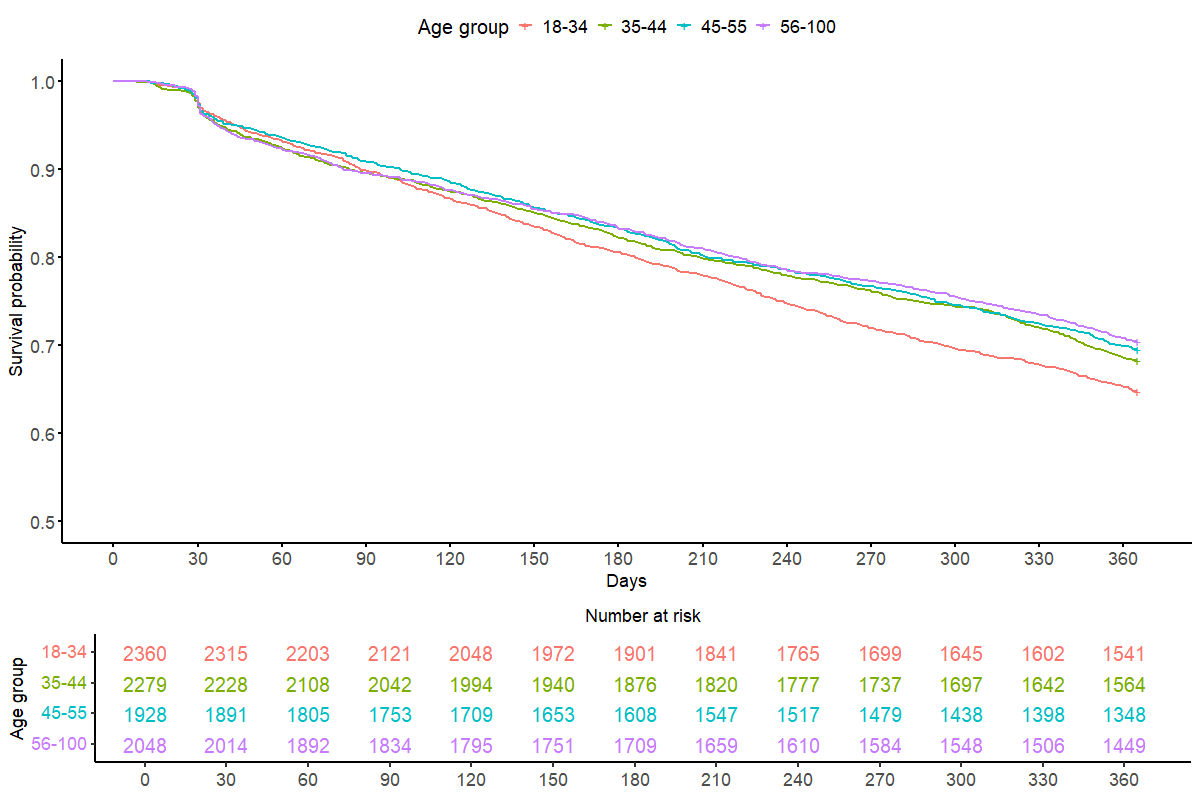
Time to device disengagement by age quartiles among 8616 connected patients over a 1-year period by age category.

Survival curves estimating patients’ probability of continued engagement with the EHR system across a 1-year period from patients’ initial day of engagement are depicted in Figures 2 and 3. These curves disaggregated the probability of survival by gender and age group. To provide a more intuitive investigation of age as a potentially impactful factor, patients’ age at sign-up was binned by quartiles. Log-rank results for these Kaplan-Meier curves indicated that (1) the probability of disengagement was not significantly different between men and women participants, and (2) patients significantly differed in survival probability by age group. Further investigation revealed that individuals significantly differed in survival probability by the respective years in which they initially linked their device and their first 7-day median step count (Figures S2 and S3 in Multimedia Appendix 1).

Overall, of the 8616 patients included in the survival analyses, 5870 (68.13%) patients remained engaged with the EHR system beyond the 365-day follow-up period for this 6-year sample from 2015 to 2022.

Figure 2 depicts the daily count of patients whose devices were still connected to the EHR framework over a 1-year period. The 2 lines within the graph depict gender, with the red line depicting participants who identify as women and the blue line depicting participants who identify as men.

Figure 3 depicts the daily count of patients whose devices were still connected to the EHR framework over a 1-year period. The red line depicts patients aged between 18 and 34 years, the green line depicts patients aged between 45 and 44 years, the blue line depicts patients aged between 35 and 55 years, and the purple line depicts patients aged between 56 and 100 years.

No significant difference in survival probability distributions was found for patients who identified as men or women (Figure 2). Figure 3 indicates a significant difference in survival probability between age quartiles. Log-rank pairwise comparisons using the Benjamini-Hochberg multiple comparisons correction indicated that patients aged between 45 and 55 years and patients aged between 56 and 100 years had a significantly different probability of disengagement as compared to patients aged between 18 and 34 years.

Table 3 displays the results of the Cox proportional hazards model created to investigate which demographic and behavioral factors significantly impacted disengagement probability within this dataset. Starting with demographics, the age quartiles covariate with ages between 18 and 34 years as reference were significant, indicating that patients aged between 35 and 44 years, between 45 and 55 years, and between 56 and 100 years were less likely to disengage from the EHR system as compared to their younger cohort. Regarding gender, no significant difference in survival trends was detected between men and women participants. Likewise, race was not significant in predicting risk of disengagement. Finally, median step counts from the first 7 days of observations per patient were entered into the model and categorized by less than 5000 steps, 5000 to 7500 steps, 7500 to 10,000 steps, and greater than 10,000 steps. Results indicated that patients who took less than a median count of 5000 steps during their first week of engagement were at a significantly higher risk of disengagement than those who took greater than 5000 steps (Table 3).

**Table 3:** Multivariable Cox proportional hazards models adjusted for age, gender, race, and baseline week median step counts among included patients connected to the electronic health record.

Covariates		Gender model	
		Hazard ratio (95% CI)	*P* value
**Age (y; reference: 18-34 y)**			
	35-44	0.87 (0.79-0.97)	.01
	45-55	0.83 (0.74-0.92)	<.001
	56-100	0.79 (0.71-0.88)	<.001
**Gender (reference: woman)**			
	Man	1.06 (0.98-1.15)	.10
**Race (reference: White)**			
	Asian	1.05 (0.93-1.19)	.39
	Black or African American	0.96 (0.84-1.09)	.56
	Other	1.02 (0.89-1.17)	.72
	Unknown	1.00 (0.88-1.14)	.95
**Median first-week step counts (steps; reference: <5000 steps)**			
	5000-7499	0.79 (0.72-0.87)	<.001
	7500-10,000	0.85 (0.75-0.96)	.01
	>10,000	0.72 (0.63-0.84)	<.001

Table 3 displays the results of the multivariable Cox proportional hazards model. This model included a categorized version of age, gender, and race and a categorized number of average steps taken within the first 7 days of device connection.

## Discussion

### Principal Findings

This study examined wearable device data collected from devices linked to our EHR system to assess patients’ long-term engagement with these technologies. This passively collected, real-world dataset is unique, as it was not associated with any specific clinical trial, spanned 6 years, and included a large and representative sample from a major metropolitan health system over a 6-year period (2015-2022). To the best of our knowledge, this is one of the first studies to examine and quantify large population nontrial user engagement from long-term wearable device data collected in-house. We observed a relatively high level of continued device engagement at 1 year, with approximately 68.13% (5870/8616) of the users still engaged at 1 year, with an overall median duration of engagement of 21 (IQR 9-38) months, indicating that the long-term use and connection of such devices is feasible in a health care population. Notably, similar levels of engagement at comparable time points have been observed in clinical trials that actively endeavor to promote sustained participant-device engagement [13]. This may be in part explained by the selection bias inherent in the voluntary, self–sign-up nature of our digital framework and therefore the curation of a subset of the population more willing to engage overall in wearable device use as previously documented in clinical trials requiring wearable device use.

We examined factors associated with device engagement and discontinued use. Demographic variables included in the Kaplan-Meier analyses yielded varying results across gender, age, and year of first device sign-up. Specifically, no statistically significant differences in engagement were noted by race as well as gender. However, our results showed that younger patients, specifically those aged between 18 and 34 years, had statistically significantly lower levels of engagement compared to older cohorts. In addition, patients who took a median of less than 5000 daily steps in their first week of engagement were at a higher risk of disengagement as compared to those who took more than 5000 daily steps, indicating that less active patients are more likely to discontinue compared to those who are more active.

Wearables provide a unique opportunity to capture longitudinal, objective activity, sleep, and other biometric data in an individual’s natural environment. Given the known benefits of physical activity and other remotely collected biometric data, it is important to monitor physical activity and sedentary and sleep behaviors over time [17,18]. These assessments were previously limited by static measures and recall biases, making it difficult to provide accurate assessments and appropriate recommendations. Therefore, wearable devices can provide a more complete picture of a person’s daily activity levels and identify the nuanced changes that occur over time. Wearable devices may also serve as potential points of intervention, which can be used to motivate healthy activity behaviors and ultimately improve health and well-being [19-21]. Importantly, their use can motivate patients to be engaged in their own health and enable personalized monitoring.

There is a current need for health care systems to improve their understanding of how engagement with wearable devices in real-world settings evolves over time and what factors predict these patterns of engagement. These engagement metrics can also be used to inform future research design, such as the inclusion of wearable devices in clinical trials, with longer-term monitoring or intervention development, where wearable devices may be powerful tools to deliver health interventions (eg, physical activity interventions) in the future. Although we observed a high level of long-term engagement, approximately 31.87% (2746/8618) of the users disengaged within a year. For long-term longitudinal trials conducted in the future, these estimates may prove to be a beneficial benchmark to compare ongoing participant engagement. Future analyses can explore the application of statistical and machine learning methods, such as cluster analysis or time-varying effect modeling, to predict engagement [15,22]. Another approach could leverage novel randomized designs, such as sequential assignment or microrandomization, to derive insights on engagement and inform the development of more dynamic and personalized strategies for promoting engagement with wearable devices [23-26]. Overall, there is a need for user and behavioral research to further advance our understanding and prediction of who disengages, when, and why.

On the policy level, understanding engagement can allow health care providers to tailor their care and identify strategies to increase the level of engagement for those who need it most. For example, the size of this voluntary cohort and its relatively low annual attrition rate highlight the scalability of large wearable device data collection. Consequently, these findings underscore the potential to monitor health and behavior trends in real time in a capacity that may generalize across the broader institution-affiliated population. The benefit of this potential reinforces the necessity for similar workflows and investigations to be replicated at other institutions. In doing so, it is hoped that similar patterns of engagement are found, reinforcing the generalizability of our findings. This effort corresponds to similar attempts to aggregate engagement data across different mobile health apps and diverse populations [15,22,27].

### Limitations

This analysis of real-world objective wearable health data from a large health care system, while insightful, has several limitations. First, our analysis set is limited to the available data and variables that were linked to the EHR; thus, it may not represent a comprehensive or complete picture of the wearable device engagement experience, and specific reasons for disengagement or device disconnection could not be obtained. Furthermore, while the user demographics are representative of those in patients seen at the institution this study’s data were collected and analyzed from, results may not be generalizable to other hospital settings and are partly explained by socioeconomic status and digital literacy [28,29]. When considering the integration of wearable devices into the health care system, it is important to take ethical considerations into account and reduce potential disparities in access and use of these technologies [30-32]. Second, device linkage may be a result of research studies that involve wearable devices, wellness initiatives, or employee health sign-ups that may impact the observed engagement in this dataset. Third, given that the dataset included metrics from multiple wearable devices, it is difficult to make comparisons across patients, as they are not standardized, although daily step counts were the most common metrics and available across all devices. In addition, we did not have control over any updates to either the EHR system or wearable device apps that resulted in loss of device connections, as observed at the end of 2021. We plan to examine the effect of system updates and other external factors, including the time-varying nature of wearable device use, on engagement in the future.

This analysis provides insights into the patterns of long-term engagement and early disengagement of wearable devices in a large health system setting. This research demonstrates how connected device data can be passively collected and leveraged to better understand health behaviors and identify factors associated with disengagement. Future analyses should focus on combining these data with clinical outcomes, such as hospitalizations and survival. These findings can also inform the evaluation and development of interventions that improve retention to enhance the clinical utility of these devices. Efforts to encourage early device integration with reminders for patients and providers to continue to link and wear devices will allow a more complete and more generalizable sample and enhance the clinical and research utility of the device data.

## Appendix

Multimedia Appendix 1 Data refinement flowchart and additional Kaplan-Meier visualizations.

## Abbreviations

EHR: electronic health record

## Data Availability

The datasets generated or analyzed during this study are available from the corresponding author on reasonable request. The shared data represents the finalized set used in the main analyses of the paper and was prepared following Figure S1 in Multimedia Appendix 1. To initiate this request, a data use agreement will need to be completed first, and a public link to the data cannot be made available. Please contact the corresponding author at their listed email address to initiate the data sharing process. The R code and data dictionary for data preparation, statistical analysis, and visualization are available upon reasonable request from the corresponding author, subject to completion of data transfer agreement forms.
