# Supplementary material for: Predicting Engagement Patterns With Connected Wearable Devices in a Health System: Survival Analysis
Source: J Med Internet Res. 2025 Sep 17;27:e78507. doi: 10.2196/78507 (PMC12489408; doi:10.2196/78507)

Supplementary Material

Supplemental Figure 1: Data Refinement Flowchart

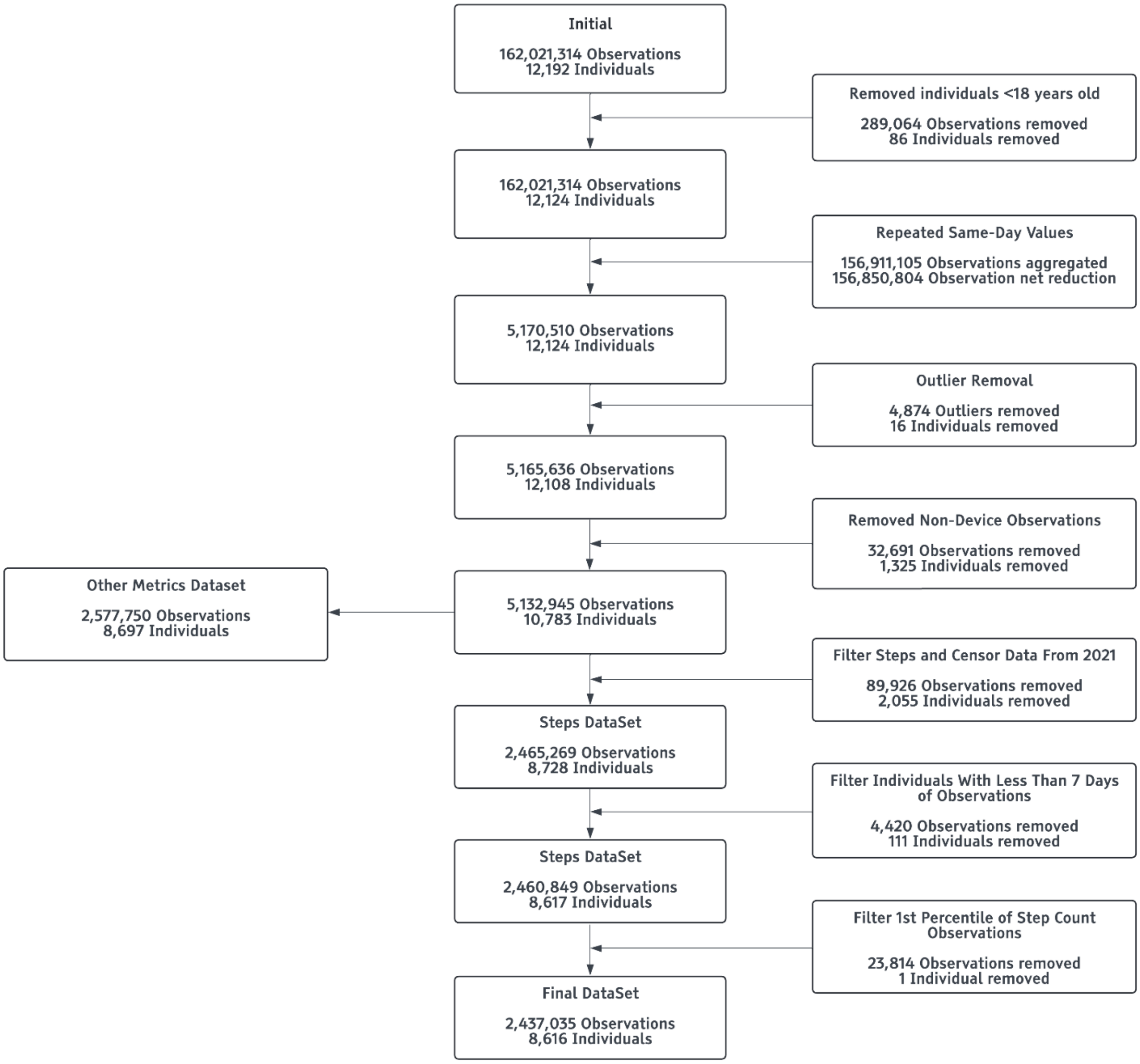

Supplemental Figure 2: 365-day Follow Up Survival by Top Two Device Type Usage

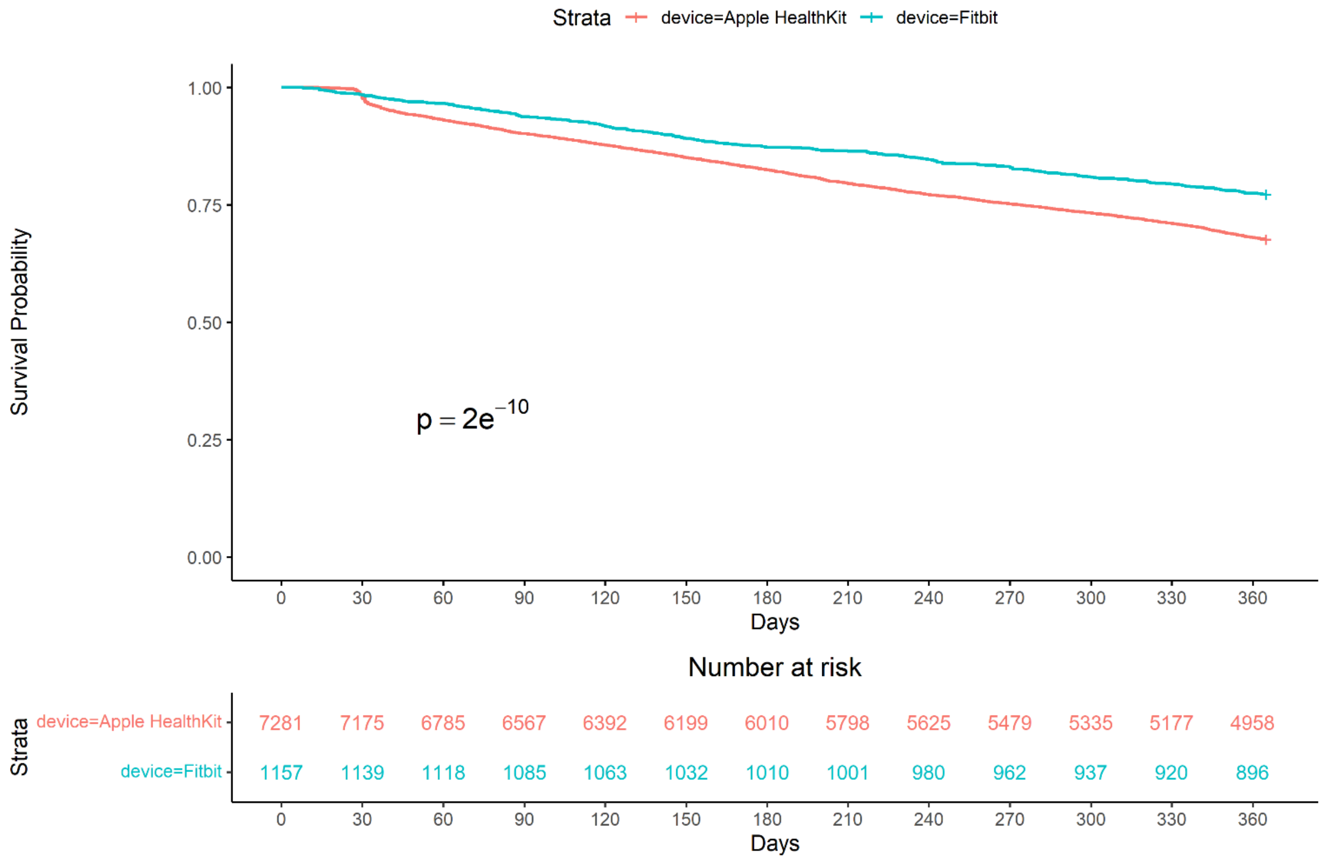

Supplemental Figure 3: 365-day Follow Up Survival by Median 7-Day Baseline Step Count

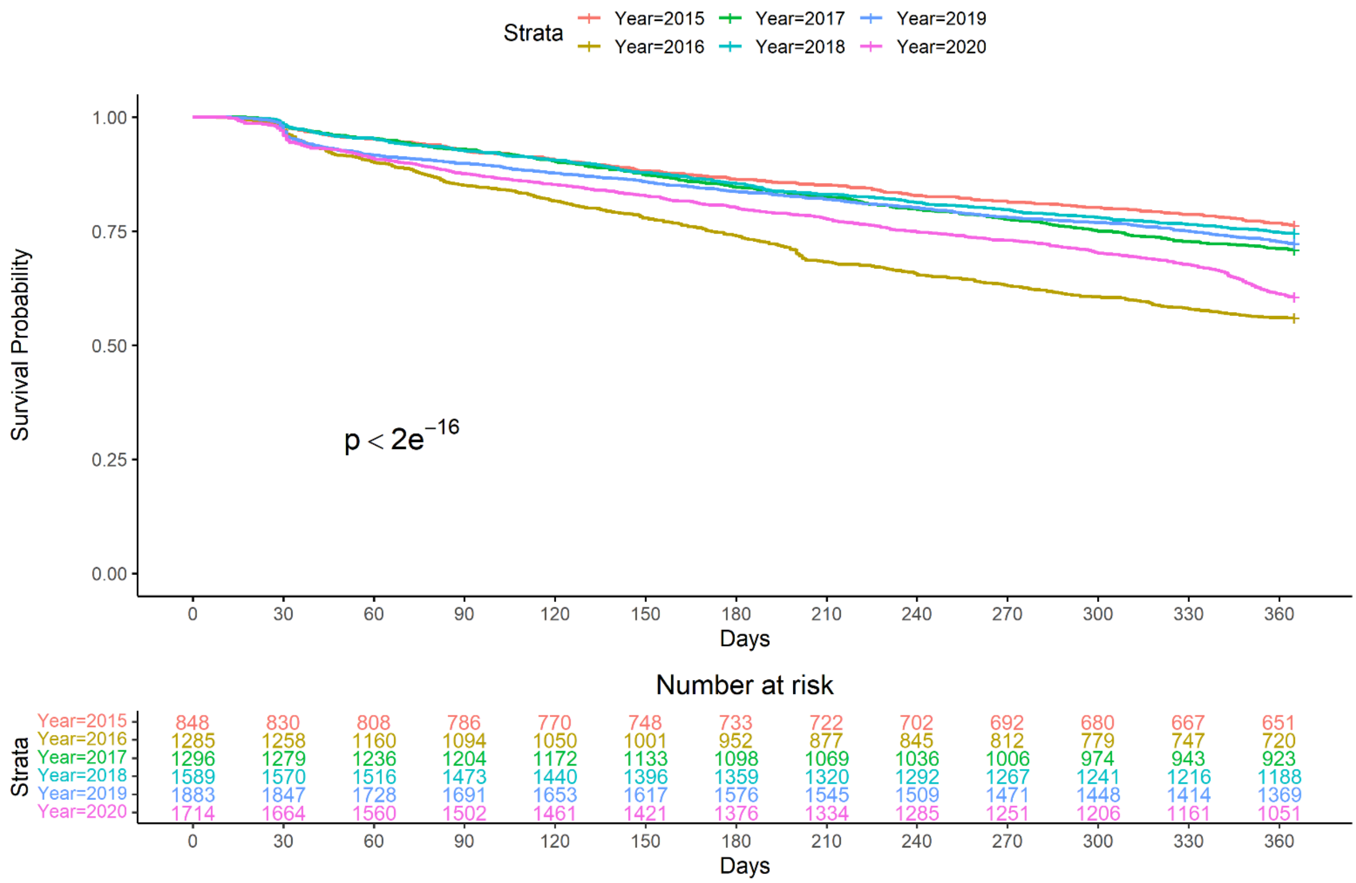

Supplement: Multimedia Appendix 1 [file jmir_v27i1e78507_app1.pdf]
